# Developing a mathematical model for the evaluation of the potential impact of a partially efficacious vaccine on the transmission dynamics of *Schistosoma mansoni* in human communities

**DOI:** 10.1186/s13071-017-2227-0

**Published:** 2017-06-17

**Authors:** Andria Stylianou, Christoforos Hadjichrysanthou, James E. Truscott, Roy M. Anderson

**Affiliations:** 1 London Centre for Neglected Tropical Disease Research, London, UK; 2 Department of Infectious Disease Epidemiology, School of Public Health, Faculty of Medicine, Imperial College London, London, UK

**Keywords:** Vaccine trials, *Schistosoma mansoni* vaccine, Partially efficacious vaccine, Mathematical modelling

## Abstract

**Background:**

There is currently no vaccine available to protect humans against infection with the schistosome digenean parasites*,* although candidate formulations for *Schistosoma mansoni* are under trial in animal models, including rodents and primates. Current strategies for the control of infection are based on mass drug administration (MDA) targeted at school-aged children of age 5 to 14 years. This approach is unlikely to eliminate exposure to infection except in settings with very low levels of transmission.

**Methods:**

A deterministic mathematical model for the transmission dynamics of the parasite is described and employed to investigate community level outcomes. The model is defined to encompass two different delivery strategies for the vaccination of the population, namely, infant (cohort) and mass vaccination. However, in this paper the focus is on vaccination delivered in a cohort immunisation programme where infants are immunised within the first year of life before acquiring infection. An analysis of the parasite’s transmission dynamics following the administration of a partially protective vaccine is presented. The vaccine acts on parasite mortality, fecundity or/and establishment.

**Results:**

A vaccine with an efficacy of over 60% can interrupt transmission in low and moderate transmission settings. In higher transmission intensity areas, greater efficacy or higher infant vaccination coverage is required. Candidate vaccines that act either on parasite mortality, fecundity or establishment within the human host, can be similarly effective. In all cases, however, the duration of protection is important. The community level impact of vaccines with all modes of action, declines if vaccine protection is of a very short duration. However, durations of protection of 5–10 years or more are sufficient, with high coverage and efficacy levels, to halt transmission. The time taken to break transmission may be 18 years or more after the start of the cohort vaccination, depending on the intensity of the transmission in a defined location.

**Conclusions:**

The analyses provide support for the proposition that even a partially efficacious vaccine could be of great value in reducing the burden of schistosome infections in endemic regions and hopefully could provide a template for the elimination of parasite transmission.

**Electronic supplementary material:**

The online version of this article (doi:10.1186/s13071-017-2227-0) contains supplementary material, which is available to authorized users.

## Background

Schistosomiasis is a parasitic disease, which infects approximately 258 million people in 78 countries and is estimated to kill 280,000 people annually [1–5]. All age groups are infected with the schistosome parasites, with children being the most vulnerable age group. The disease induced by infection is typically chronic and debilitating, with the severity dependent on parasite burden [1, 6].

The primary form of public health control of schistosomiasis is mass drug administration (MDA) using praziquantel. However, MDA is inadequate to provide long term protection against the parasite and repeated treatment must be administered in endemic regions at intervals of 1 to 2 years [7, 8]. Past infection does not protect against reinfection post-drug treatment. Immunological responses to parasite antigens can be detected in the human host but they do not appear to generate protective levels of acquired immunity to prevent reinfection. MDA in endemic regions is having a beneficial impact on the burden of disease caused by the human schistosome parasites, but the drug availability as well as the logistical problems of getting treatment to remote communities, has prevented progress on controlling this infection in many regions of the world. Ideally, a vaccine is needed and much effort has been directed towards improving understanding of the human immune responses to infection and why the parasite is able to successfully re-infect individuals after drug treatment [9, 10].

Currently, there is no prophylactic or therapeutic vaccine available for human use to protect against the schistosome parasites (or indeed other helminth infections). However, experiments in mice, rodents, hamsters and baboons infected with *S. mansoni*, have shown partial prophylactic and anti-fecundity efficacies using various candidate formulations, including recombinant Sm-p80 protein and DNA priming followed by boosting with various parasite proteins [6, 11–15]. Recombinant protein with the Sm-p80 antigen is the leading vaccine candidate at present [16, 17]. A human schistosome vaccine that provides some protection may be possible, although efficacy will be measured in partial effects *via* reducing adult parasite survival, fecundity and establishment [6]. This may be due to the candidate vaccines inducing immunity that act to reduce but not eliminate adult parasite establishment and growth within the immunized hosts.

Published studies on schistosome transmission dynamics under the impact of various interventions have largely focused on the effect of MDA and snail control on the mean adult parasite burden, assuming a constant human population size with a fixed age structure [18–23]. Some mathematical model-based studies have looked at the potential impact of vaccines, but the models have been based on the dynamics of the human population described by a Susceptible-Vaccinated-Infected-Recovered (SVIR) framework [24–26]. This type of model construct may not be appropriate for examining the impact of a partial efficacious vaccine that acts differentially on parasite longevity, fecundity and establishment in the human host. The experimental studies referenced earlier in animal models of various candidate vaccines, suggest effects on all three population processes in the vaccinated mammalian host. In these circumstances, a macro-parasite model framework is ideally required, which could track changes in parasite burden in the human host. Past model development of this type is built on the early studies of Macdonald and Anderson & May, and has included full age structure of the human host population, sexual mating probabilities, density dependent fecundity, acquired immunity, and aggregated parasite distributions per human host (described by the negative binomial probability model) [27, 28]. Individual-based stochastic models have also been developed to describe the transmission dynamics of the human schistosome parasites and the impact of MDA [29, 30]. Mathematical model development for the study of the transmission dynamics of schistosome infections in humans has recently been reviewed by Anderson and colleagues [30].

In this paper, based on recent pre-clinical studies in primates [6, 14, 15], we describe the development of a simple deterministic mathematical model which details the dynamics of the human host and adult parasite populations, to assess the impact of a potential vaccination programme that is applied to a community. The model has a general framework such that it can mirror different vaccine delivery strategies, namely infant and mass immunisation. If mass vaccination takes place across all age classes in an area of endemic infection, then uncertainties arise surrounding the effects of immunising already infected individuals and those with past experience of infection. In this paper, the generic model is presented but analyses are focused on the vaccination at birth strategy. The model is constructed to show the potential vaccine effect on either adult worm mortality, fecundity or establishment. In subsequent papers more complex models will be examined, including full age structure and individual based stochastic frameworks.

The classic macro-parasite transmission dynamics model of Anderson & May [27] is extended to investigate the potential effect of a partially efficacious vaccine based on the properties defined by early experimental studies in animal models which include reducing the parasite’s life expectancy, fecundity and rate of establishment in the mammalian host [6, 11–15]. We incorporate into the model sexual mating probabilities, negative binomial distributions of adult worms per human host and density dependence in egg output. The main aim is to provide some information on the potential of a partially efficacious vaccine for *S. mansoni* to interrupt transmission community wide, prior to the conduct of expensive phase I, II and III trials in humans.

## Methods

A deterministic mathematical model (including probability terms for the parasite distribution within the human host and the mating probability) is developed to facilitate investigation of the potential impact of different community based vaccination programmes using a vaccine candidate with defined properties at the individual, and concomitantly, the community level. The model includes representation of the dynamics of the human host population and can represent two different intervention delivery scenarios; infant (cohort) or mass vaccination. The candidate vaccine is assumed to act either on adult worm survival, fecundity or establishment in the human host.

Analytical studies of model properties are presented, where functions can be derived for key quantities such as the critical level of vaccine coverage required to interrupt transmission.

### The effects of vaccination on the adult worm population dynamics

A human helminth vaccine would ideally contain antigens to stimulate host immunological responses that affect three factors. These are the per capita mortality rate of the adult worms, *σ*, the number of eggs being produced per female worm per unit of time, *λ*, and the rate at which the cercarial larvae are able to infect and grow to sexual maturity within the human host on contact, *β*. The efficacies of a vaccine affecting the above three factors are denoted by *v*_1_ ∈ [0, 1] , *v*_2_ ∈ [0, 1] and *v*_3_ ∈ [0, 1], respectively. Hence, *v*_1_ = *v*_2_ = *v*_3_ = 0 is the case where the vaccine has no efficacy, while *v*_1_ = *v*_2_ = *v*_3_ = 1 is the case where the vaccine is 100% effective. After a vaccine administration, the rates *σ* , *λ* and *β* become *σ* ^′^, *λ* ^′^ and *β* ^′^, respectively, where:

1 $$ {\sigma}^{\prime }=\left(\frac{1}{1-{v}_1}\right)\sigma,\ 0\le {v}_1<1 $$

2 $$ {\lambda}^{\prime }=\left(1-{v}_{\kern.1em 2}\right)\kern.1em \lambda,\ 0\le {v}_2\le 1 $$

3 $$ {\beta}^{\prime }=\left(1-{v}_3\right)\kern.1em \beta,\ 0\le {v}_3\le 1 $$

### Host and parasite population dynamics

**Fig. 1:**
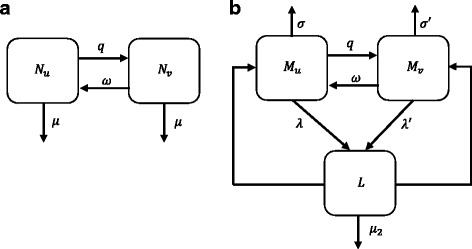
Schematic diagrams representing the host (**a**) and the parasite (**b**) population dynamics

An individual in the host population is defined as belonging to one of two groups, either the vaccinated or the unvaccinated, denoted by *N*_*v*_ and *N*_*u*_, respectively. In the case of a cohort immunisation programme a proportion *p* of infants aged from 0 to 1 year old are chosen randomly to receive a single dose of vaccine, yearly. On the other hand, mass vaccination is applied to randomly chosen individuals at a per capita rate *q* per year. The selection procedure does not take into account the sex, age, socioeconomic status or other characteristics of the individuals, but instead we assume a homogeneous population. Vaccine-induced immunity is lost at a rate *ω*, i.e. $$ \tau =\raisebox{1ex}{$1$}\!\left/ \!\raisebox{-1ex}{$\omega $}\right. $$ is the average duration of vaccine protection. When the vaccine protection wanes the vaccinated individuals move back to the unvaccinated group. The parasites have a different life-cycle, depending on whether or not they grow within an immunised individual. Thus, the parasitic population has similar dynamics to the host population. The variables *M*_*u*_ and *M*_*v*_ represent the average worm burden inhabited unvaccinated and vaccinated hosts. The models of the human and the parasite populations are represented schematically in Fig. 1. Note that both populations are dynamically connected *via* the contact of the human with the cercaria released by infected snails, represented by the transmission coefficients, *β* or *β* ^′^ (depending on which vaccination state the host belongs to). The parameters influencing the host and the parasite populations are described in Table 1.

**Table 1:** Parameter values for the host and parasite (*S. mansoni*) populations and key vaccination parameters, used in the evaluations of the models as summarised in [31]

Variable/Parameter	Interpretation	Values
*N*	Host population size	–
*M*	Mean number of adult worms	Model dependent
*L*	Environmental reservoir	–
*μ* ^b^	Human host death rate	1/50 yrs^-1^
σ^b^	Parasite mortality rate	1/4 yrs^-1^ [27]
*μ*_2_^b^	Free-living larvae mortality rate	365/7 yrs^-1^ [27]
*λ* ^b^	Egg production rate per female worm	0.14 [31]
*β* ^b^	Rate of contact between human and infective stages	0.63^a^ yrs^-1^ [31]
*ψ*	Flow of infectious material into the environment	–
*k*	Negative binomial clumping parameter	0.24 [34]
*γ*	Density dependence fecundity parameter	0.0006/female worm [34]
*p*	Proportion of infants vaccinated per unit of time	[0,1]
*q* ^b^	Rate of mass vaccination	–
*ω* ^b^	Rate of loss of vaccine-induced immunity per unit of time (vaccine duration of protection: $$ \tau =\raisebox{1ex}{$1$}\!\left/ \!\raisebox{-1ex}{$\omega $}\right.\Big) $$	[0,∞)
*v*_1_	Vaccine efficacy with an effect on worm life expectancy	[0,1]
*v*_2_	Vaccine efficacy with an effect on worm fecundity	[0,1]
*v*_3_	Vaccine efficacy with an effect on worm establishment	[0,1]

^a^The value of *β* was estimated using the weighted average of the different age groups from [31]

^b^Per capita rates per unit of time

As it is customary with models of this form, the dynamics of the life-cycle stages outside the human host (miracidia, infected snail hosts and cercariae) are assumed to be turning over on a very fast time scale (hours, days or weeks) compared to the adult parasite life cycle (4–6 years) within humans. Therefore, the dynamics of the infective stages are adjusted to the steady state value and we focus on the temporal dynamics of the mature worm and egg output dynamics [29].

Note that it is assumed that the vaccine has no impact on the host mortality rate, *μ*. In the Additional file 1: Figure S1 records the age and sex pyramids for Malawi in 2016. The graphs show an almost constant mortality rate by age with a mean life expectancy of approximately 50 years. We also consider that the immunised individuals receive the vaccine-induced immunity benefits instantaneously - no time delays in the induction of immunity are taken into account. Throughout the paper the assumption that the human death rate, the rate of loss of vaccine-induced immunity and the continuous vaccination rate are age- and time-independent holds.

Vaccination programmes will not only affect the mean intensity of infection within the vaccinated individuals but will also have an indirect impact on the unvaccinated host population (herd immunity effects) due to changes in both egg output and worm burden in the vaccinated individuals that reduces the overall transmission within the entire community. This is the reason that the host population is split into two groups, vaccinated and unvaccinated people. The dynamics of the two vaccination groups is described by the following system of differential equations:

4 $$ \frac{d{N}_u}{ d t}=- q{N}_u+\omega {N}_v - \mu {N}_u, $$

5 $$ \frac{d{N}_v}{ d t}= q{N}_u-\omega {N}_v-\mu {N}_v. $$

It should be noted that the total population size is *N*(*t*) = *N*_*u*_(*t*) + *N*_*v*_(*t*), where *N*(*t*) = *e* ^-*μt*^. The equations shown above do not include a vaccination at birth term, but this term will turn up through the initial conditions of new births entering the host population. If a fraction *p* is vaccinated at birth, then *N*_*v*_(0) = *p*.

The above generic model can be reduced to represent the following model framework:

- Model 1: Vaccination of a proportion *p* of infants within the age range [0, 1), (*q* = 0).
- Model 2: Vaccination of the general population at a per capita rate *q* per year (*p* = 0).

The dynamics of the worm burden within the unvaccinated and the vaccinated host population and the environmental reservoir, denoted by *M*_*u*_, *M*_*v*_ and *L*, respectively, are described by the following system of equations:

6 $$ \frac{d{M}_u}{ d t}= L{\beta}_u-\left(\mu +\sigma \right){M}_u- q{M}_u+\omega {M}_v $$

7 $$ \frac{d{M}_v}{ d t}= L{\beta}_v - \left(\mu +{\sigma}^{\prime}\right){\kern.1em  M}_v+ q{M}_u - \omega {M}_v $$

8 $$ \frac{dL}{dt}=\psi \left(\lambda {M}_u+{\lambda}^{\prime }{M}_v\right) - {\mu}_2 L $$

where *β*_*u*_ and *β*_*v*_, are defined as:

9 $$ {\beta}_u=\frac{\beta \left(\mu +\omega - p\mu \right)}{\left(\omega + q+\mu \right)} $$

10 $$ {\beta}_v=\frac{\beta^{\prime}\left( q+ p\mu \right)}{\left(\omega + q+\mu \right)} $$

The parameter *ψ* characterises the flow of infectious material into the environment. The derivation of eqs. (9) and (10) can be found in the Additional file 2.

Note that in the above equations we did not take into account the density dependence on egg output and the mating probability functions.

The mean parasitic load within a community can instinctively be defined as the weighted average of the worms within the vaccinated and the unvaccinated individuals of a community, i.e.

11 $$ M=\left(1- p\right){M}_u+ p{M}_v $$

All the results in the next section, consider the mating probability and the density dependence function and they are solved numerically. In this case, eq. (8) becomes:

12 $$ \frac{dL}{dt}=\psi \left(\lambda {M}_u F\left({M}_u\right)+{\lambda}^{\prime }{M}_v F\left({M}_v\right)\right)-{\mu}_2 L $$

The function *F*(*M*) is a product of density dependence and mating probability times the normalised host population of each group. The exact definition of this is given by Anderson & May [27] and can be found in the Additional file 2. In this study we assume that the negative binomial distributed parasites are monogamous and have a fixed value for the aggregation parameter, *k*.

### Basic and effective reproductive numbers (*R*_0_, *R*_*e*_)

The basic reproductive number, *R*_0_, is defined as the average number of female offspring produced per female adult worm, that survive to reproductive maturity in the absence of density dependent constraints on parasite population growth [27]. This is a crucial quantity which determines whether the parasite will spread and persist within the host population. The parasite persistence criterion is *R*_0_ > 1 in the absence of density dependent constraints, and if *R*_0_ falls below unity the parasite population cannot maintain itself.

A definition for the effective reproductive number can be constructed using the master equation for the probabilities of being in the two states, vaccinated or unvaccinated, using the transition rates. The output equation is:

13 $$ {R}_e=-\frac{\psi}{\mu_2}{\boldsymbol{\varLambda}}^T{\boldsymbol{M}}^{-1}\boldsymbol{B}, $$

where,

$$ \boldsymbol{\Lambda} =\left(\lambda, {\lambda}^{\prime}\right),\kern1em \boldsymbol{M}=\left(\begin{array}{cc}-{\mu}_u& \omega \\ {} q& -{\mu}_v\end{array}\right),\kern1em \boldsymbol{B}=\left(\genfrac{}{}{0pt}{}{\beta_u}{\beta_v}\right), $$

with *μ*_*u*_ = *μ* + *σ* + *q* and *μ*_*v*_ = *μ* + *σ* ^′^ + *ω*. A detailed derivation of eq. (13) is provided in the Additional file 2.

In the relationship above *Μ* is the matrix describing the transition rates.

Substituting the above vectors and the matrix in (13) we obtain:

14 $$ {R}_e=\frac{\psi}{\mu_2\left({\mu}_u{\mu}_v- q\omega \right)}\left[\lambda, \left({\mu}_v{\beta}_u+\omega {\beta}_v\right),+,{\lambda}^{\prime },\left( q{\beta}_u+{\mu}_u{\beta}_v\right)\right]. $$

In the absence of any interventions *R*_*e*_ is reduced to *R*_0_ given by:

15 $$ {R}_0=\frac{\psi \lambda \beta}{\mu_2\left(\mu +\sigma \right)}. $$

Throughout the results section, we explore predicted impacts for a range of *R*_0_ values. In broad terms a low transmission setting has values in the range of (1, 1.4], a medium transmission setting has values in the range [1.5, 2.5) and anything over 2.5 is a high transmission setting. These observations are based on the estimation of *R*_0_ values for *S. mansoni* from field studies in villages with low, medium and high transmission intensities that record a full cross sectional age intensity profile (eggs per gram (epg) measures) [30, 31].

Let ${R}_0^v$ be the effective reproductive number when the infant vaccination coverage is 100%, i.e. *p* = 1 and under the assumption that the vaccine is imperfect.

The effective reproductive number in an infant immunisation programme can be written as the sum of the effective reproductive number in the unvaccinated and fully vaccinated populations, weighted by the proportion of unvaccinated and vaccinated infants, respectively:

16 $$ {R}_e=\left(1- p\right){R}_0+ p{R}_0^v, $$

The derivation of eq. (16) is given in the Additional file 2.

### Critical infant vaccination coverage, *p*_c_

The prime interest is in the proportion of people that have to be successfully vaccinated in order to achieve the interruption of transmission, i.e. in the critical vaccination level, *p*_*c*_, so that R_e_ < 1. Reformulating eq. (16) for *p* and setting the restriction R_e_ < 1, we have:

17 $$ {p}_c=\frac{1-\frac{1}{R_0}}{1-\frac{R_0^v}{R_0}}. $$

The numerator of equation (17) gives the critical vaccination level of a perfect vaccine, with 100% efficacy on mortality, fecundity and establishment (*v*_1_ = *v*_2_ = *v*_3_ = 1), and a lifelong protection (*ω* = 0).

It should also be noted that the value calculated from equation (17) does not take into account the density dependent effects on parasite fecundity and, most importantly, the mating probability at low parasite burdens where both males and females must be in the same host to produce viable offspring to sustain transmission. The numerical calculations of the Results section, include both the density dependent and the mating probability functions [23].

### Parameters uncertainty

There is some uncertainty surrounding key parasite population biology parameter assignments (as reviewed in [30]), especially the expected lifespan of the adult worms in the human host. Current estimates of this parameter, which are in the range of 3.5 to 8 years, are unlikely to be refined in the near future so we analyse the impact of a vaccine using a consensus set of parameter values [31]. Moreover, we recognise that results are sensitive to changes in this particular parameter. Other key parameters, such as the age group dependent infection rates, are derived using Markov Chain Monte Carlo (MCMC) methods by fitting models to observed age intensity and age prevalence profiles for *S. mansoni* [27, 30, 31].

In the assignment for parameter values for the vaccine efficacy, we consider a wide range of options for the effect on all three parasite population parameters (establishment in the host and growth to maturity, adult worm life expectancy and fecundity). Experiments in animal models of the candidate vaccine labelled Sm-p80, suggest that vaccination acts on all three worm population variables, but most effectively on the establishment of female and male worms [6, 14, 15]. Vaccination may also affect parasite lifespan for those parasites who do grow to maturity in the immunised host, but the effects are not quantified as yet. In the case of fecundity, again those worms who manage to establish in the vaccinated hosts do seem to have a reduced fecundity, but again quantitative measures are not available at present.

As far as the duration of protection is concerned, little is known of how long protection against worm establishment will last, although experiments in mice suggest that antibodies against vaccine antigens persist for long periods [6, 11]. Ideally, experiments need to be performed to look at this issue but these need to be carried out over long time intervals, perhaps up to 10 to 20 years. This is an important aspect and the implications are examined by making various assumptions about the average expectancy of protection under the assumption of a constant decay rate of immunity with a half-life of *τ*.

As noted earlier, the importance of the duration of protection will depend to a large extent on the prevailing background mortality in the human population. If, for example, the death rate over the infant and school aged child age classes is high, as it is in many poor regions of the world, many vaccinated individuals will die before moving into the teenage age classes in an infant cohort-based programme. As such, even with high vaccine coverage soon after birth, the overall proportion of immunised people in the total population may never reach a high level assuming both vaccinated and unvaccinated individuals have similar rates of death. For this reason, the importance of the duration of protection will depend on this early life background human mortality rate. Almost exponential decay distributions of numbers of people by age are observed in some poor regions, which is the assumption embedded in the model (a constant rate of mortality, *μ*, independent of age).

## Results

The analytical solutions of the system of equations (4)–(5) and (6)–(7) can be found in the Additional file 2.

### Infant immunisation with lifelong protection for varying efficacies

In this sub-section, we make the optimistic assumption that the benefit of immunisation is lifelong, i.e. *ω* = 0.

**Fig. 2:**
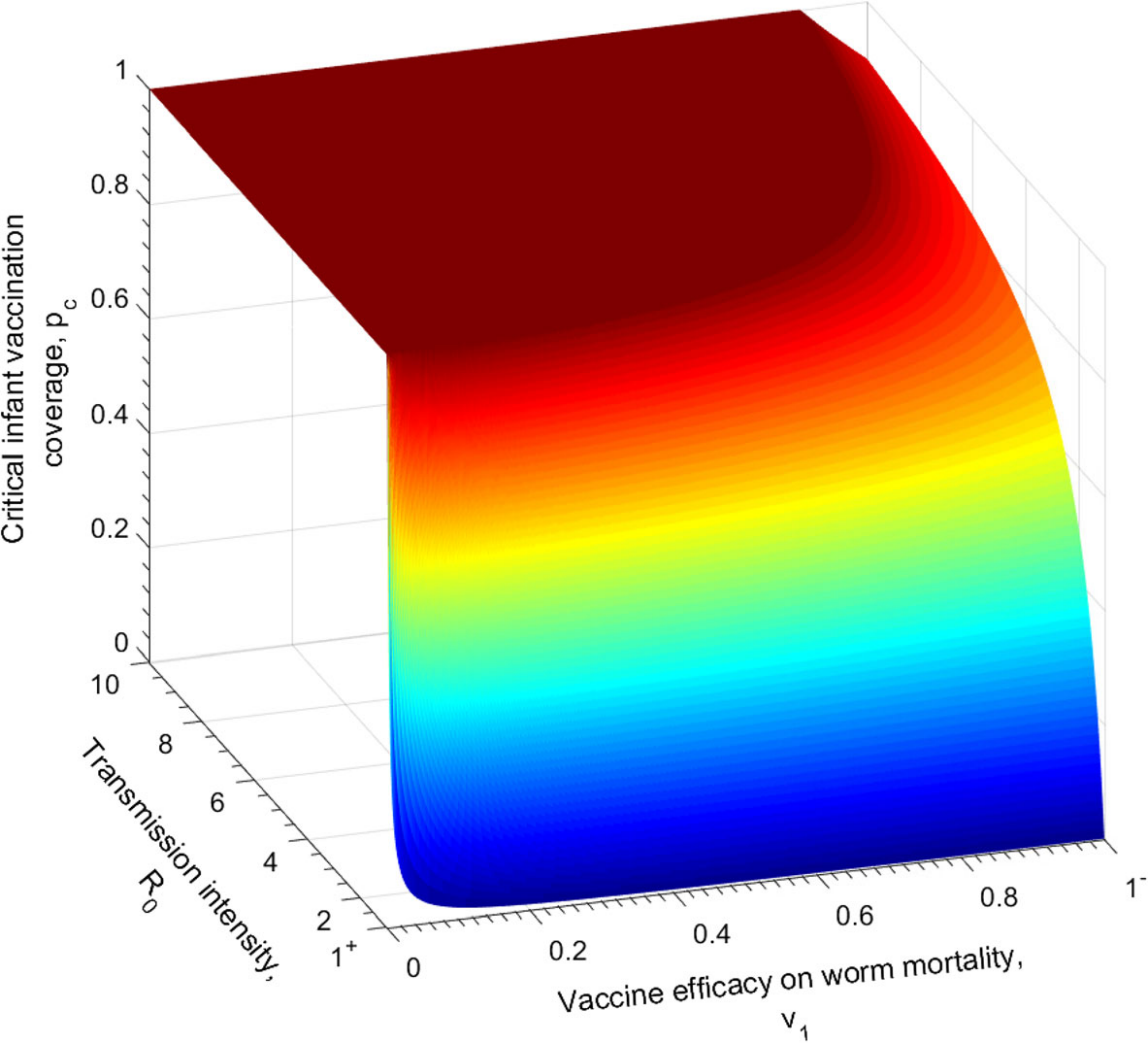
The relationship between the critical infant vaccine coverage required to break transmission for different *R*_0_ values, and the efficacy of a vaccine with an impact on increasing the mortality of the adult worm. The duration of vaccine protection is lifelong (*ω* = 0). The other vaccine impact parameters are set as *v*_2_ = 0 , *v*_3_ = 0, i.e. no effect on parasite fecundity and establishment. The parasite population parameters are defined in Table 1

Figure 2 shows the dependence of the critical infant vaccination level, *p*_*c*_, on the efficacy of the vaccine that reduces the adult worm life expectancy and on the transmission intensity in a defined location, *R*_0_, such that the transmission of the parasite is interrupted, i.e. *R*_*e*_ < 1.

The pattern portrayed in Fig. 2 shows that for moderate (which are the most usual) transmission settings (*R*_0_ = 1.1−2.5), a vaccine efficacy of 63% reduction in adult worm survival at an infant annual coverage of 100% will be able to interrupt transmission. For high transmission settings, for example *R*_0_ = 4, a vaccine that provides full efficacy, 100% for a minimum of 75% infant coverage will be able to break transmission.

Note that for the parameter space where the surface reaches a plateau, all infants must be vaccinated in order the disease to be eliminated. In some cases, even this is not sufficient and more frequent vaccinations or booster vaccine doses will be required within a year to reduce *R*_*e*_ < 1. As we will examine later in this section, the time taken to get to the point of transmission interruption, will be long in a cohort immunisation programme simply because it takes time to build up herd immunity.

**Fig. 3:**
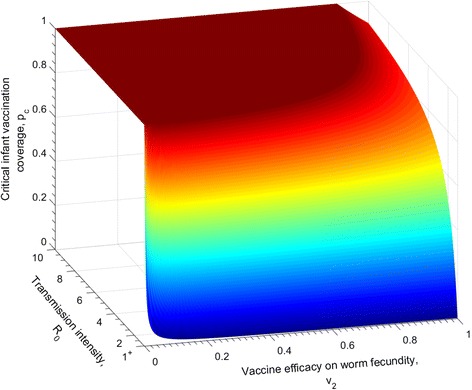
The relationship between the critical infant vaccine coverage required to break transmission for different *R*_0_ values, and the efficacy of a vaccine with an impact on reducing the per capita fecundity of adult worms. The duration of vaccine protection is lifelong (*ω* = 0). The other vaccine impact parameters are set as *v*_1_ = 0 , *v*_3_ = 0, i.e. no effect on parasite survival and establishment. The parasite population parameters are defined in Table 1

The relationship between *p*_*c*_ and the efficacy of a vaccine that impacts fecundity is displayed in Fig. 3 for different *R*_0_ values. The pattern is very similar to the one presented in Fig. 2. In medium transmission settings stopping transmission by infant vaccination with a moderate vaccine efficacy of 60% reduction in worm fecundity requires 100% coverage. Again, for high transmission settings *R*_0_ > 4, a vaccine with full efficacy will break transmission when the vaccination coverage is at least 75%.

**Fig. 4:**
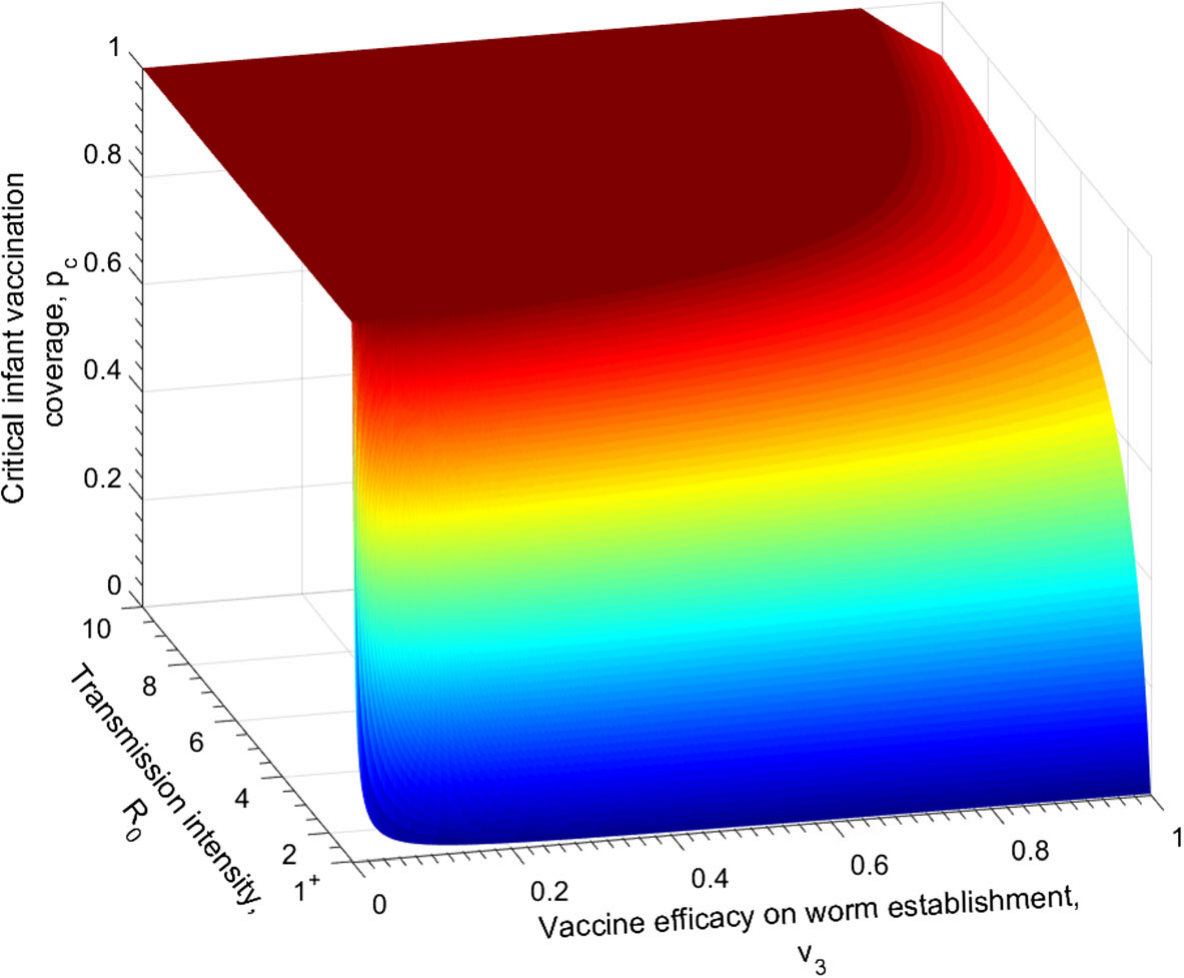
The relationship between the critical infant vaccine coverage required to break transmission for different *R*_0_ values, and the efficacy of a vaccine with an impact on reducing the worm transmission. The duration of vaccine protection is lifelong (*ω* = 0). The other vaccine impact parameters are set as *v*_1_ = 0 , *v*_2_ = 0, i.e. no effect on parasite survival and fecundity. The parasite population parameters are defined in Table 1

A vaccine that has a potential effect on the adult worm establishment has similar impacts to those that influence the worm survival and fecundity, as the plotted surface shows in Fig. 4. Moderate *R*_0_ values in medium vaccine efficacy levels, 60%, will halt transmission if all infants are being immunised every year.

The similarities in the predictions of the impact of the three modes of vaccine action (reducing parasite fecundity, life expectancy and establishment in the human host) in Figs. 2, 3 and 4, relates to the fact that all three parameters impact the magnitude of the effective reproductive number in a similar linear manner.

### Duration of vaccine protection

In the previous subsections, the duration of protection was taken to be lifelong. Now we relax this assumption and the associated impact of vaccine duration on the critical vaccination coverage, *p*_*c*_, is explored.

**Fig. 5:**
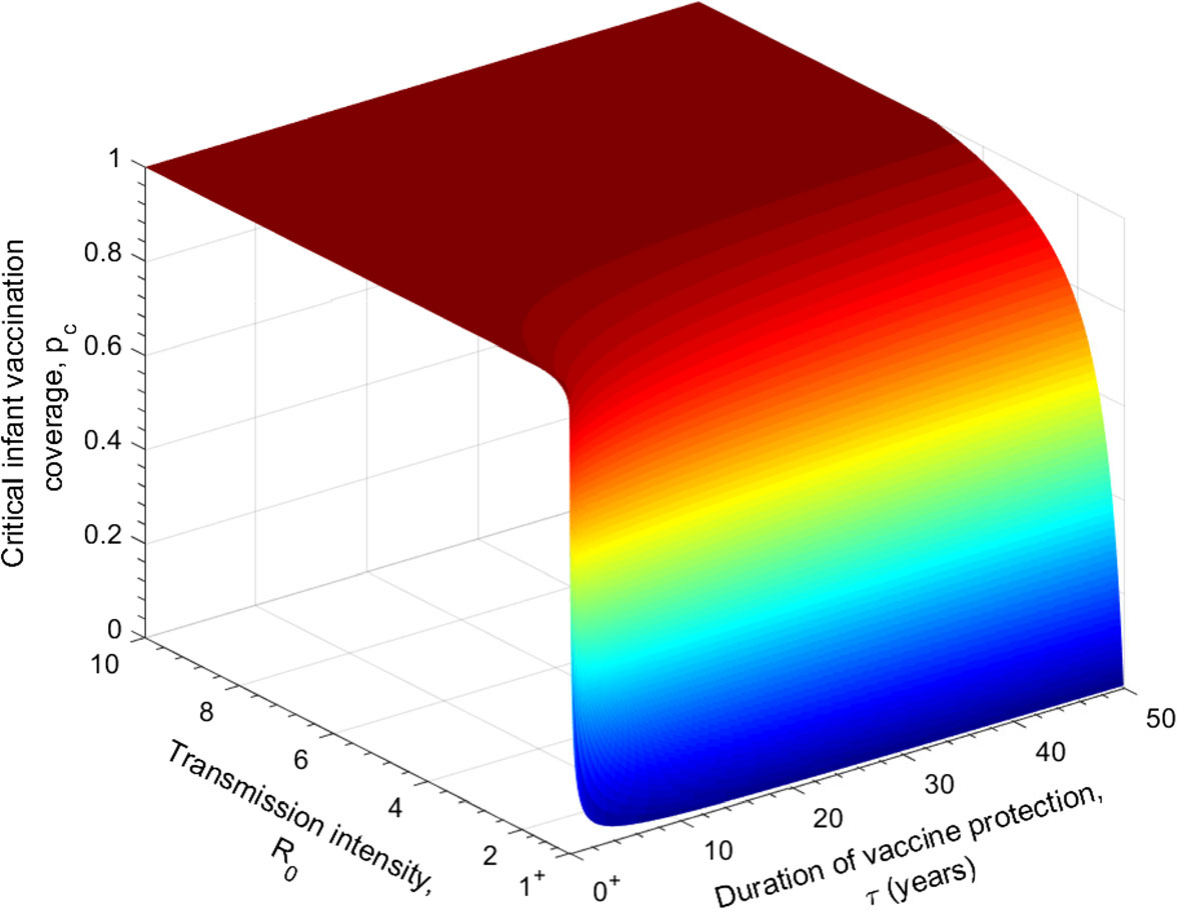
The relationship between the proportion of the population that must be immunised per year to halt transmission, as a function of the transmission intensity in a defined area and the average duration of protection, $$ \tau =\raisebox{1ex}{$1$}\!\left/ \!\raisebox{-1ex}{$\omega $}\right. $$ *,* in years. Vaccine efficacies are set as: *v*_3_ = 0.80 , *v*_1_ = *v*_2_ = 0

Figure 5 shows that vaccine protection duration is critical after 5 years in a moderate transmission environment. If a vaccine provides 80% efficacy in the reduction of worm establishment and a protection for less than 5 years then little benefit arises in increasing the value of the proportion of the population that require vaccination each year. The predicted pattern is very much influenced by the background mortality of the human host population. As described earlier, the assumption of a constant mortality rate, independent of age, implies continual loss of those vaccinated. This in turn creates difficulties in maintaining herd immunity.

**Fig. 6:**
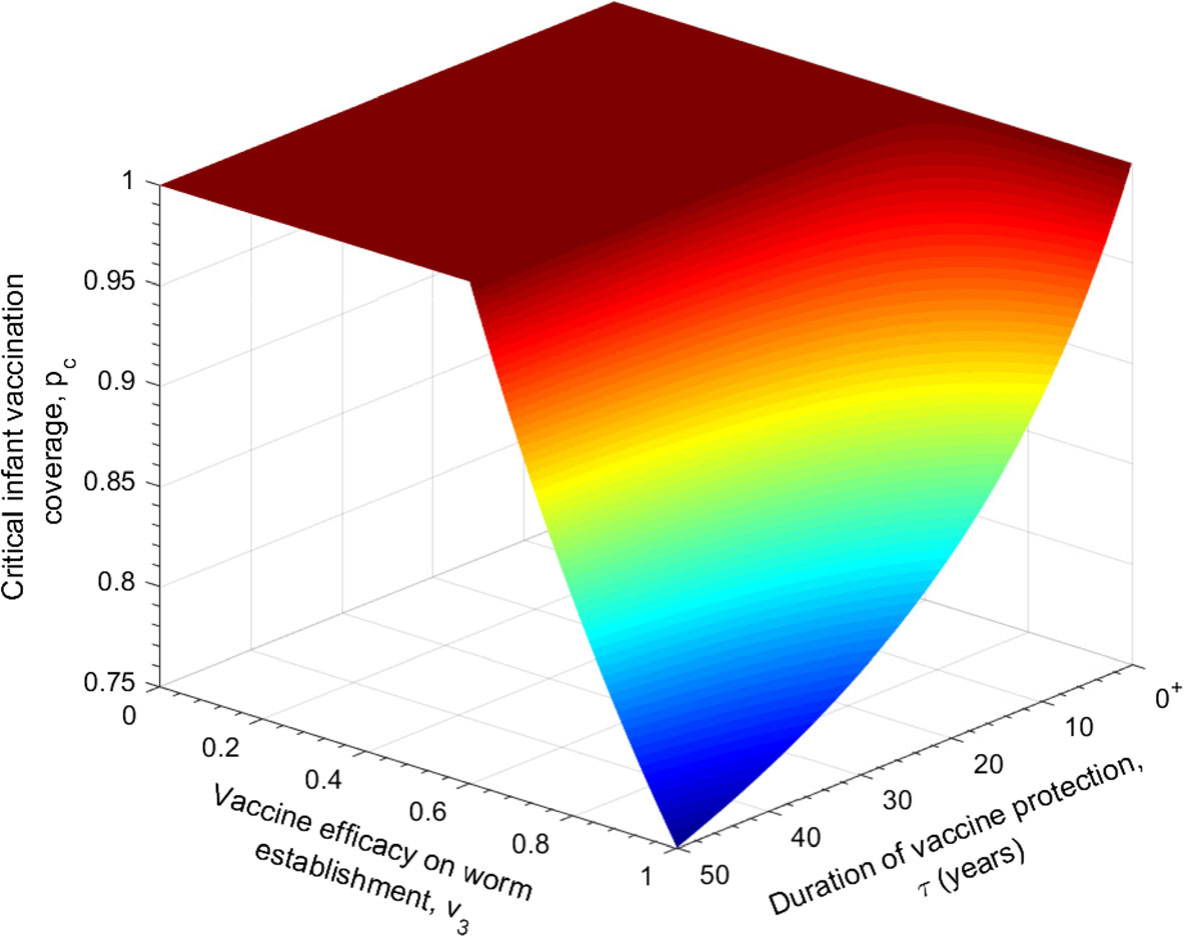
The relationship between the critical vaccination proportion of infants, with the vaccine efficacy on worm establishment and the average duration of protection. The other parameters are defined as: *R*_0_ = 2.5 , *v*_1_ = *v*_2_ = 0

The critical vaccination level increases significantly for medium efficacies on worm establishment and *R*_0_ = 2.5, if a potential vaccine provides less than 10 years of protection, as illustrated in Fig. 6. In addition, a vaccine with 100% efficacy in the reduction of worm establishment that provides lifelong immunity (at least 50 years), requires 75% of newly borns to be immunised to break the transmission of the parasite.

Table 2 records the sensitivity of the critical infant vaccination coverage in the variation in the rate of loss of vaccine induced immunity, *ω*, for Model 1. As mentioned before, the duration of protection has limited effect on *p*_*c*_ as long as it is below 5 years (Fig. 5). On the other hand, a considerable reduction of the proportion of infants that have to be vaccinated to halt transmission may occur when the duration of protection is long (decades).

**Table 2:** Impact of intervention when vaccine protection duration varies for different *R*_0_ values

Average duration of vaccine protection (years)	Critical infant vaccination coverage, *p*_*c*_ (%)
1	99
2	98
5	97
10	94
20	91
50 (Lifelong)	85

The parameters have the following fixed values: *v*_1_ = 0 , *v*_2_ = 0 , *v*_3_ = 0.80 , *R*_0_ = 2.5

### Vaccine impact on the mean worm burden

In community-based vaccination programmes, vaccine efficacy and coverage may not reach high enough levels to halt transmission. Stopping transmission is of course not the only desired outcome, and lower efficacies can have a very substantial impact on the mean worm burden and hence morbidity.

**Fig. 7:**
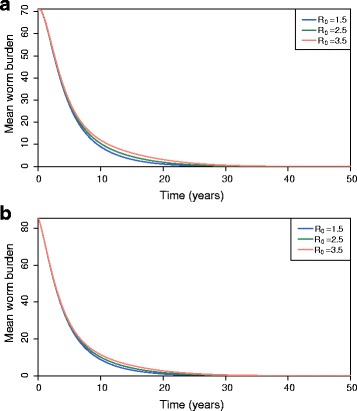
The impact of the transmission setting, *R*_0_ *,* for Model 1 on the temporal trend in the mean worm burden *M*, with 80% vaccine efficacy on parasite establishment only, i.e. *v*_1_ = 0 , *v*_2_ = 0 , *v*_3_ = 0.80*,* for (**a**) *p* = 70% and (**b**) *p* = 85%. The starting value for the mean worm burden is *M*_0_ = 100 per host and the vaccine protection is assumed to be lifelong, i.e. *ω* = 0

Figure 7a, b shows the equilibrium worm burden for different transmission settings achieved by a vaccine that impacts only adult parasite establishment in the human host with an efficacy of 80% and an infant coverage of 70% and 85%, respectively. Table 3 records the numerical mean worm burden values at equilibrium 50 years after vaccination for low, medium and high transmission settings. Note that worm elimination can be achieved even with *R*_0_ = 3.5 and 70% immunisation coverage of infants, but high efficacy levels, 80%, in worm establishment are required.

**Table 3:** The equilibrium worm burden, *M* ^∗^ for different *R*_0_ values with the vaccine efficacy set as: *v*_1_ = 0*, v*_2_ = 0*, v*_3_ = 0.80 and the vaccination coverage as *p* = 70% and *p* = 80%

Transmission intensity (*R*_0_)	Equilibrium mean worm burden (*M* ^∗^), *p* = 0.70	Equilibrium mean worm burden (*M* ^∗^), *p* = 0.85
1.5	3.51 × 10^-4^	3.41 × 10^-4^
2.5	9.71 × 10^-4^	8.76 × 10^-4^
3.5	3.33 × 10^-3^	2.47 × 10^-3^

The duration of vaccine protection is assumed to be lifelong, ω = 0. In the absence of vaccination, the endemic mean worm burden is *M*_0_ = 100 worms per host

**Fig. 8:**
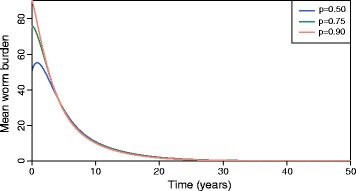
The impact of infant vaccine coverage, *p*, for Model 1 on the temporal trend in the mean worm burden, with 80% efficacy of a vaccine having an impact on parasite establishment, i.e. *v*_1_ = 0 , *v*_2_ = 0 , *v*_3_ = 0.80, and *R*_0_ = 2.5. The starting value for the mean worm burden used is *M*_0_ = 100 per host

Cohort immunisation takes time to impact the level of herd immunity within infants even for high vaccination coverage levels. Figure 8 shows the temporal trends in the mean worm burden after the introduction of vaccination. For a 50% coverage, and assuming a 80% vaccine efficacy acting on parasite’s establishment in a moderate transmission setting, *R*_0_ = 2.5, it takes approximately 21 years to eliminate the worm population. In part, this is related to the impact of human mortality on the build-up of herd immunity (the mortality rate in infant age is very high, and hence many vaccinated infants die, and in part by the slow dynamic time scale of the system set by adult worm life expectancy, set as 4 years. This is the reason we obtain a small increase in the mean worm burden up to 1 year after the intervention is introduced. The numerical equilibrium values of worm burden for various proportions of infant vaccination are given in Table 4.

**Table 4:** The equilibrium worm burden, *M* ^∗^ for Model 1 at time *t* = 50 years for different vaccination levels, with the vaccine efficacy parameters set as: *v*_1_ = 0*, v*_2_ = 0*, v*_3_ = 0.80 and the transmission intensity constant: *R*_0_ = 2.5

Vaccination coverage, *p* (%)	Equilibrium worm burden (*M* ^∗^)
50	7.40 × 10^-4^
75	9.66 × 10^-5^
90	7.80 × 10^-6^

The duration of vaccine protection is assumed to be lifelong, *ω* = 0. In the absence of vaccination, the endemic mean worm burden is *M*_0_ = 100 worms per host

## Discussion

A vaccine to protect against schistosome infection is ideally required to eliminate the parasite in endemic regions. MDA alone is effective in preventing morbidity in children, but breaking transmission will require high coverage levels in children and adults over many decades [30, 31]. This study describes a mathematical model for the potential effect of a schistosome vaccine if applied to the human host population, under two different vaccine delivery strategies, namely, infant and mass vaccination. The mathematical model explores the vaccination coverage required to achieve transmission elimination. In these calculations, the possible impact of a partially efficacious vaccine is considered. The vaccine acts to reduce the parasite’s life expectancy, fecundity, and adult worm establishment within the human host.

An important factor that the developed model investigates is the duration of vaccine-induced immunity, which determines the required time to break the transmission of the parasite. To measure the duration of protection requires the repeated challenge of immunised animals over say 1, 5 and 10 years. In practise, such experiments on the duration of protection may not be possible for justifiable animal welfare reasons, including regulations which restrict the period of time an infected animal can be left untreated. As such, duration of protection may need to be tested in humans, if progression through phase I and phase II clinical trials is successful. This would entail the careful design of phase IV trials to measure reinfection rates, as has been the case for the candidate malaria vaccine [32].

The period taken to break parasite transmission following an infant vaccination programme could be possibly greatly reduced by applying a mass vaccination strategy across all age classes. In this circumstance, the safety of vaccinating those already infected, if a potential mass immunisation programme is applied, is of obvious importance and needs to be established *via* clinical trials. Previous studies have shown that MDA can reduce up to 87% the worm burden [33]. Thus, theoretically, it may be best to treat with MDA first across all age classes and then immunise. Other unknowns include, the safety and impact of vaccination on uninfected people (post treatment of praziquantel) who have had short, or long, past experience of infection, given the potential generation of good immunological responses that can be generated in previously infected individuals.

In addition, the long average timescale set for adult worm life expectancy (3.5–8) years that will negate a strong influence of changes on an annual basis and the precise nature of the density dependence effect are considered limitations of our model. Little improvement on the understanding of these processes is expected in the near future.

This paper has focused on the impact of infant vaccination. However, the mathematical model framework is general in form, and allows mass vaccination to be explored. Future publications will model combination of interventions (MDA and vaccination) and different delivery options in both an age structured hybrid model and a full individual based stochastic model.

The real challenge though, is in finding a financial model that will pay for the development of such candidates to progress *via* costly trials in humans to test safety, efficacy and community based impact, to vaccine production and manufacture for servicing resource poor settings. Despite this rather pessimistic note, what is encouraging is the fact that vaccine candidates of reasonable efficacy in primates can be developed which have the potential to interrupt transmission in endemic regions of the world.

## Conclusions

A series of general conclusions emerge from the analyses. First, a vaccine with moderate efficacy of 60% will, according to our mathematical model, interrupt transmission in communities with low and moderate transmission. For high transmission settings, higher vaccine efficacies are required to interrupt transmission or multiple booster vaccine doses each year may be necessary. Second, the analysis suggests that a candidate vaccine that impacts either on worm establishment, worm fecundity or adult parasite survival in the human host is almost equally beneficial. Experiments in mice, rodents and baboons suggest that all three factors may be affected by the lead vaccine candidate (Sm-p80 protein), where reduced female worm growth in immunised hosts acts to decrease fecundity, and perhaps survival as well. The ongoing experiment on baboons will give more detail on the vaccine impact on parasite’s establishment, growth, and fecundity within the immunised animals. Third, with an infant based vaccination programme, the benefits of immunisation will take some time to become apparent due to the slow build-up of herd immunity in a cohort vaccination programme. Our model shows that breaking transmission in even low intensity transmission areas, may take 18 years or more of medium to high infant coverage.

Fourth, the conclusions outlined above depend on the duration of protection generated by one or a short course of vaccination. If duration is less than a few years, repeated immunisation may be necessary per year for breaking transmission. The mathematical model we have developed suggests that the duration does not need to be life-long to achieve elimination of the disease. An average duration of protection of the order of 5–10 years is adequate to ensure good community based impact at moderate to good efficacy and good coverage levels.

## Additional files

Additional file 1: Figure S1.Population pyramid by age and sex of Malawi from the US Bureau of Population and Census database (https://www.census.gov/population/international/data/idb/region.php?N=%20Results%20&T=12&A=separate&RT=0&Y=2016&R=−1&C=MI). The plots show exponential decay in population size by age, with a mean life expectancy $$ \left(\raisebox{1ex}{$1$}\!\left/ \!\raisebox{-1ex}{$\upmu $}\right.\right) $$ for the total population of approximately 50 years. (TIFF 105 kb)

Additional file 2:Analytical solutions of the host population dynamics. Analytical solutions of the parasite population dynamics. Derivation of equations of the transmission functions. Density dependence and mating probability functions. Derivation of the effective reproductive number, *R*_*e*_. Derivation of *R*_*e*_ formula in terms of *R*_0_ and ${R}_0^v$. (DOCX 29 kb)

## Abbreviations

Epg: Eggs per gram
MDA: Mass drug administration
