# Supplementary figures and images for: Developing a mathematical model for the evaluation of the potential impact of a partially efficacious vaccine on the transmission dynamics of Schistosoma mansoni in human communities
Source: Parasit Vectors. 2017 Jun 17;10:294. doi: 10.1186/s13071-017-2227-0 (PMC5474049; doi:10.1186/s13071-017-2227-0)

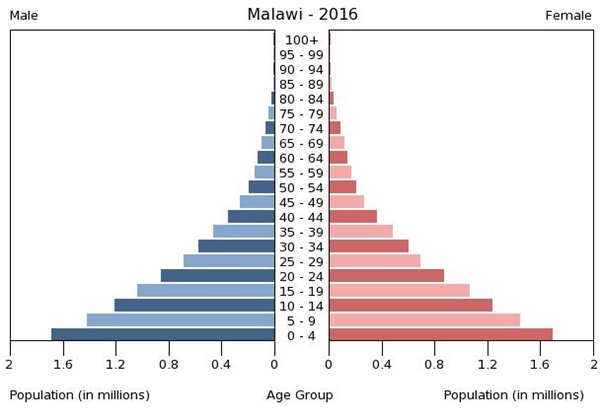

Supplement: Supplementary file 1 — Population pyramid by age and sex of Malawi from the US Bureau of Population and Census database (https://www.census.gov/population/international/data/idb/region.php?N=%20Results%20&T=12&A=separate&RT=0&Y=2016&R=−1&C=MI). The plots show exponential decay in population size by age, with a mean life expectancy $$ \left(\raisebox{1ex}{$1$}\!\left/ \!\raisebox{-1ex}{$\upmu $}\right.\right) $$1μ for the total population of approximately 50 years. (TIFF 105 kb) [file 13071_2017_2227_MOESM1_ESM.tif]
