# Supplementary material for: Developing a mathematical model for the evaluation of the potential impact of a partially efficacious vaccine on the transmission dynamics of Schistosoma mansoni in human communities
Source: Parasit Vectors. 2017 Jun 17;10:294. doi: 10.1186/s13071-017-2227-0 (PMC5474049; doi:10.1186/s13071-017-2227-0)
Supplement: Supplementary file 2 — Analytical solutions of the host population dynamics. Analytical solutions of the parasite population dynamics. Derivation of equations of the transmission functions. Density dependence and mating probability functions. Derivation of the effective reproductive number, R e. Derivation of R e formula in terms of R 0 and ${R}_0^v$R0v. (DOCX 29 kb) [file 13071_2017_2227_MOESM2_ESM.docx]

**Additional file 2**

Analytical solutions of the host population dynamics

The analytical solutions of the system of equations (4)-(5) given in the main text, are:

$$N_{u}\left( t \right)=\left( \frac{\omega}{\omega+q} \right)e^{-\mu t}-\left( p-\frac{q}{\omega+q} \right)e^{-\left( \mu+\omega+q \right)t},$$

$$N_{v}\left( t \right)=\left( \frac{q}{\omega+q} \right)e^{-\mu t}+\left( p-\frac{q}{\omega+q} \right)e^{-(\mu+\omega+q)t}.$$

Analytical solutions of the parasite population dynamics

If we assume that the environmental reservoir is at equilibrium:

$$L^{*}=\frac{1}{\mu_{2}}\psi(\lambda M_{u}+\lambda^{'}M_{v})$$

The initial conditions for the worm burden dynamics, given by equation (6) and (7) in the main text, are given by the product of the equilibrium parasite load within an individual before vaccination, $M_{0}$, and the proportion vaccinated and unvaccinated individuals:

$$M_{u}\left( t=0 \right)=M_{0}N_{u}\left( t=0 \right)=\left( 1-p \right)M_{0},$$

$$M_{v}\left( t=0 \right)=M_{0}N_{v}\left( t=0 \right)=pM_{0}.$$

The analytical solutions for the worm burden, excluding the mating probability and the density dependence function are given by:

$$M_{u}\left( t \right)=k_{1}\left[ Ae^{r_{1}t}\left( r_{1}-k_{2} \right)+Be^{r_{2}t}(r_{2}-k_{2}) \right],$$

$$M_{v}\left( t \right)=Ae^{r_{1}t}+Be^{r_{2}t}.$$

The constants are:

$$k_{1}=\frac{\mu_{2}}{\beta_{v}\psi\lambda+q\mu_{2}}, k_{2}=\frac{\beta_{v}\psi\lambda^{'}}{\mu_{2}}-\left( \mu+\sigma^{'}+\omega\right),$$

$$A=pM_{0}-\frac{1}{\left( r_{1}-r_{2} \right)}\left[ {pM}_{0}\left( r_{1}+\mu+\sigma^{'}+\omega-q\left( \frac{1}{p}-1 \right) \right)-L_{0}\beta_{v} \right],$$

$$B=\frac{1}{(r_{1}-r_{2})}\left[ {pM}_{0}\left( r_{1}+\mu+\sigma^{'}+\omega-q\left( \frac{1}{p}-1 \right) \right)-L_{0}\beta_{v} \right],$$

where $r_{1}$ and $r_{2}$ are the roots of a quadratic equation:

$$r_{1,2}=\frac{-b\pm\sqrt{b^{2}-4ac}}{2a},$$

with coefficients:

$$a=1,$$

$$b=\left( \mu+\sigma+q \right)-\frac{\beta_{u}\psi\lambda}{\mu_{2}}-k_{2},$$

$$c=\frac{\beta_{u}\psi\lambda k_{2}}{\mu_{2}}-\frac{\beta_{u}\psi\lambda^{'}}{k_{1}\mu_{2}}-k_{2}\left( \mu+\sigma+q \right)-\frac{\omega}{k_{1}}.$$

Derivation of equations of the transmission functions

Transmission functions for the two groups, unvaccinated and vaccinated, are derived by multiplying the respective transmission coefficients, $\beta$ and $\beta^{'}$, by the analytical solutions of the host population dynamics.

$$\beta_{u}=\beta\int_{t=0}^{\infty} N_{u}\left( t \right) dt=\frac{\beta(\mu+\omega-p\mu)}{\mu(\omega+q+\mu)},$$

$$\beta_{v}=\beta^{'}\int_{t=0}^{\infty} N_{v}\left( t \right) dt=\frac{\beta^{'}(q+p\mu)}{\mu(\omega+q+\mu)}.$$

Here we assumed that that the mortality rate, the rate of loss of vaccine-induced immunity and the continuous vaccination rate are age- and time-independent.

It should also be mentioned that the derived equations for $\beta_{u}$ and $\beta_{v}$ are different from equations (9) and (10) represented in the main text. This is because in the main text the normalised form is presented, after dividing by $\frac{1}{\mu}$.

Density dependence and mating probability functions

The function $F\left( M \right)$ is used to denote the product of the mating probability, $\varphi(M,k)$, and the density dependence, $f(M)$, functions. For the defined functions below we assume that the parasites follow a negative binomial distribution, are monogamous and have a fixed aggregation parameter value, $k$.

$$F\left( M \right)=f\left( M \right)\varphi\left( M,k \right)N$$

where

$$f\left( M \right)=\left[ 1+\frac{M(1-z)}{k} \right]^{-(k+1)},$$

$$\varphi\left( M,k \right)=1-\frac{\left( 1-\gamma\right)^{1+k}}{2\pi}\int_{0}^{2\pi} \frac{1-\cos\vartheta}{\left( 1+\gamma\cos\vartheta\right)^{1+k}}d\vartheta, \gamma=\frac{M}{(M+k)}$$

It should be noted that the product both $f(M)$ and $\varphi(M,k)$ functions give an individual based outcome. In order to obtain the community level outcomes we have to multiply by the host population.

Derivation of the effective reproductive number, $R_{e}$

The master equations for the probabilities of worms being in the two states, vaccinated and unvaccinated, using the transition rates are:

$M_{u}\to M_{u} :1-\left( \mu+\sigma+q \right)dt=1-\mu_{u}dt$, where $\mu_{u}=\left( \mu+\sigma+q \right)$

$$M_{u}\to M_{v} :qdt$$

$M_{v}\to M_{v} :1-\left( \mu+\sigma^{'}+\omega\right)dt=1-\mu_{v}dt$, where $\mu_{v}=\left( \mu+\sigma^{'}+\omega\right)$

$$M_{v}\to M_{u} :\omega dt$$

The master equations in a matrix form:

$$\frac{d}{dt}\binom{M_{u}}{M_{v}}=\left( {-\mu_{u} \atop q} {\omega\atop-\mu_{v}} \right)\binom{M_{u}}{M_{v}}=\boldsymbol{M}\binom{M_{u}}{M_{v}}$$

Since the eigenvalues of $\boldsymbol{M}$ are non-zero, then it is a non-singular matrix and thus invertible. Hence, we can write the above in the following form:

$$\boldsymbol{M}^{-1}\frac{d}{dt}\binom{M_{u}}{M_{v}}=\binom{M_{u}}{M_{v}}$$

Using the eigen-decomposition of the matrix $\boldsymbol{M}$ we get:

$$\boldsymbol{M=VE}\boldsymbol{V}^{-1},$$

and thus the inverse of $\boldsymbol{M}$ is given in the form:

$$\boldsymbol{M}^{-1}=\boldsymbol{V}\boldsymbol{E}^{-1}\boldsymbol{V}^{-1},$$

where $\boldsymbol{E}$ is a diagonal matrix, whose elements are the eigenvalues of $\boldsymbol{M}$, and $\boldsymbol{V}$ is the eigenvector matrix, given by:

$$\boldsymbol{V}=\left( {\omega\atop e_{1}+\mu_{u}} {\omega\atop e_{2}+\mu_{u}} \right), \boldsymbol{E}=\left( {e_{1} \atop0} {0 \atop e_{2}} \right) .$$

Because $E$ is a diagonal matrix, then we have:

$$\boldsymbol{E}^{-1}=\left( {\frac{1}{e_{1}} \atop0} {0 \atop\frac{1}{e_{2}}} \right)$$

The eigenvalues of $\boldsymbol{M}$ are given by:

$$e_{1,2}=\frac{-(\mu_{u}+\mu_{v})\pm\sqrt{{(\mu_{u}-\mu_{v})}^{2}+4q\omega}}{2}$$

Let the egg productions of the two states be: $\boldsymbol{\Lambda}=\left( \lambda, \lambda^{'} \right).$ The contribution of eggs from an established worm, $Q,$ will be:

$$Q=-\psi\int_{t=0}^{\infty} \boldsymbol{\Lambda}\binom{M_{u}}{M_{v}}\left( t \right)dt = -\psi\boldsymbol{\Lambda}\int_{t=0}^{\infty} \boldsymbol{M}^{-1}\frac{d}{dt}\binom{M_{u}}{M_{v}}\left( t \right)dt$$

$$Q=-\psi\boldsymbol{\Lambda}\boldsymbol{M}^{-1}\left. \binom{M_{u}}{M_{v}} \right|_{\Pi}=-\psi\boldsymbol{\Lambda}\boldsymbol{M}^{-1}\boldsymbol{\Pi}$$

where $\Pi=\left( \pi_{u},\pi_{v} \right)$ are the initial conditions of $(M_{u},M_{v})$.

If we put $n$ eggs into the environment, on average, $\frac{n(\beta_{u}+\beta_{v})}{\mu_{2}}$ will establish. The probability split between the two types of hosts will be:

$$\binom{\pi_{u}}{\pi_{v}}=\frac{1}{(\beta_{u}+\beta_{v})}\binom{\beta_{u}}{\beta_{v}}.$$

Hence, $R_{e}$ will be:

$$R_{e}=-\frac{\psi}{\mu_{2}}\boldsymbol{\Lambda}^{T}\boldsymbol{M}^{-1}\boldsymbol{B,}$$

(1)

for

$$\boldsymbol{B}=\binom{\beta_{u}}{\beta_{v}}.$$

Derivation of $R_{e}$ formula in terms of $R_{0}$ and $R_{0}^{v}$

$R_{0}^{v}$ can be defined by setting $p=1$ and $q=0$ in the definition of the effective reproductive number, $R_{e}$, given by equation (1):

$$R_{0}^{v}=\frac{\omega}{(\omega+\mu)}R_{0}+\frac{\lambda^{'}\beta^{'}\mu\psi}{\mu_{2}(\omega+\mu)(\omega+\mu+\sigma^{'})}+\frac{\lambda\beta^{'}\mu\psi\omega}{\mu_{2}(\omega+\mu)(\mu+\sigma)(\omega+\mu+\sigma^{'})}$$

(2)

Expanding equation (1):

$$R_{e}=\frac{R_{0}(\omega-p\mu+\mu)}{(\omega+\mu)}+\frac{\lambda\beta^{'}\mu\psi\omega}{\mu_{2}(\omega+\mu)(\mu+\sigma)(\omega+\mu+\sigma^{'})}p+\frac{\lambda^{'}\beta^{'}\mu\psi}{\mu_{2}(\omega+\mu)(\omega+\mu+\sigma^{'})}p$$

(3)

Thus, plugging (2) in (3) we obtain:

$$R_{e}=\left( 1-p \right)R_{0}+pR_{0}^{v}$$
